# Congenic Mapping and Allele-Specific Alteration Analysis of *Stmm1* Locus Conferring Resistance to Early-Stage Chemically Induced Skin Papillomas

**DOI:** 10.1371/journal.pone.0097201

**Published:** 2014-05-20

**Authors:** Kazuhiro Okumura, Megumi Saito, Eriko Isogai, Ikuo Miura, Shigeharu Wakana, Ryo Kominami, Yuichi Wakabayashi

**Affiliations:** 1 Department of Carcinogenesis Research, Division of Experimental Animal Research, Chiba Cancer Center Research Institute, Chiba, Chiba, Japan; 2 Technology and Development Team for Mouse Phenotype Analysis, Japan Mouse Clinic, Riken Bioresource Center, Tsukuba, Ibaraki, Japan; 3 Department of Molecular Genetics, Graduate School of Medical and Dental Sciences, Niigata University, Niigata, Niigata, Japan; Ohio State University Medical Center, United States of America

## Abstract

Genome-wide association studies have revealed that many low-penetrance cancer susceptibility loci are located throughout the genome; however, a very limited number of genes have been identified so far. Using a forward genetics approach to map such loci in a mouse skin cancer model, we previously identified strong genetic loci conferring resistance to early-stage chemically induced skin papillomas on chromosome 7 with a large number of [(FVB/N×MSM/Ms)×FVB/N] F1 backcross mice. In this report, we describe a combination of congenic mapping and allele-specific alteration analysis of the loci on chromosome 7. We used linkage analysis and congenic mouse strains to refine the location of *Stmm1* (*Skin tumor modifier of MSM 1*) locus within a genetic interval of about 3 cM on proximal chromosome 7. In addition, we used patterns of allele-specific imbalances in tumors from F1 backcross and N10 congenic mice to narrow down further the region of *Stmm1* locus to a physical distance of about 5.4 Mb. To gain the insight into the function of *Stmm1* locus, we carried out a long term BrdU labelling experiments with congenic mice containing *Stmm1* locus. Interestingly, we observed a decrease of BrdU-LRCs (Label Retaining Cells) in a congenic strain heterozygous or homozygous for MSM allele of *Stmm1*. These results suggest that *Stmm1* responsible genes may have an influence on papillomagenesis in the two-stage skin carcinogenesis by regulating epidermal quiescent stem cells.

## Introduction

Identification of the specific genetic variants responsible for increased susceptibility to familial or sporadic cancers remains an important but challenging goal with major implications for the prediction of individual cancer risk, as well as for improved strategies for prevention or targeted therapy [1]–[4]. Present approaches to detect low-penetrance tumor-susceptibility alleles in humans involve association studies using DNA samples from hundreds or thousands of cancer patients, and an equal number of well-matched controls. Such studies are plagued by confounding factors such as population heterogeneity, weak effects, and genetic interactions, and require a very large number of cases and controls to reach statistical significance [5]–[7]. For many complex-trait diseases, including cancer, low-penetrance susceptibility alleles account for a very small proportion of the total cancer risk [8], leading to considerable discussion of the best approaches to discover the majority of disease-causing alleles in human populations.

For these reasons, complementary gene mapping and validation approaches including cross-species comparisons using animal models are required to identify genes that modify disease phenotypes, including the risk of developing cancer [9]–[11]. Exploiting the resistance of *M. spretus* to the two-stage skin carcinogenesis model involving 7,12-dimethylbenz(a)anthracene (DMBA) initiation and subsequent promotion with 12-*O*-tetradecanoylphorbol-13-acetate (TPA), 15 skin tumor susceptibility loci, *Skts1-15*, were identified in an interspecific F1 backcross [(*NIH/Ola*×*M. spretus*)×*NIH/Ola*] using QTL analysis [12], [13]. In addition to the *Skts* series, several other skin tumor modifier loci were identified using commonly used inbred strains or wild-derived strains. *Skts-fp1-3* were identified in a cross between the wild-derived inbred strain PWK and FVB/N [14]. *Skts-fp1* was also identified in a study involving a cross between a wild-derived outbred stock of *Mus musculus castaneus* and FVB/N [15]. We previously reported mapping of *Stmm1* and *2* (*Skin tumor modifier of MSM*), which confer resistance to skin tumor development on mouse chromosome 7 in a cross between the resistant Japanese wild-derived inbred strain MSM/Ms and the susceptible strain FVB/N [16]. In the present study, we used interval-specific congenic mouse strains to refine the location of *Stmm1* within a genetic interval spanning approximately 3 cM on proximal chromosome 7. In addition, we used patterns of allele-specific imbalances in tumors from F1 backcross and N10 congenic mice to further refine the location of *Stmm1*. High frequency of either MSM allele loss or FVB allele gain was detected in the region corresponding to physical distance of about 5.4 Mb. To gain the further insight into the function of *Stmm1* locus, we carried out BrdU chase experiments with congenic lines containing *Stmm1*. As a result, we observed a decrease of BrdU-LRCs in congenic strains heterozygous or homozygous for the MSM allele of *Stmm1* on proximal chromosome 7. These results suggest that gene(s) located within the *Stmm1* locus may have an influence on papillomagenesis in the two-stage skin carcinogenesis by regulating epidermal quiescent stem cells.

## Results

### A Strong Papilloma Resistance Locus, *Stmm1 (Skin Tumor Modifier of MSM*), on Proximal Chromosome 7

**Figure 1:**
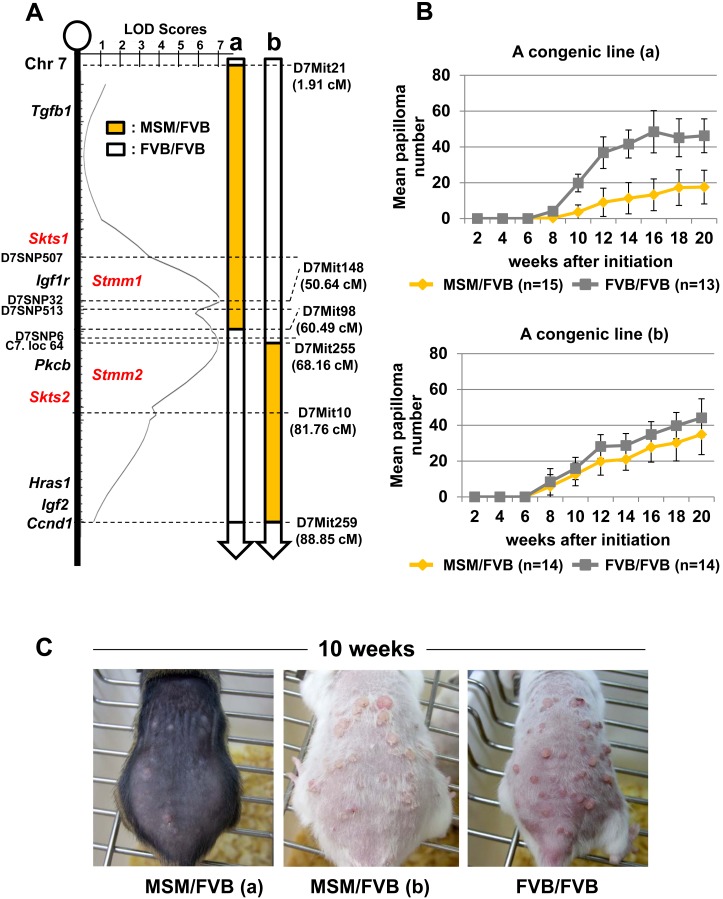
Genetic linkage map and papilloma incidence in congenic lines (a) and (b) on mouse chromosome 7. (A) Two significant linkage peaks, *Stmm1* and *2* were mapped in the previous report [16]. Two orange and white bars represents two congenic lines (a) and (b). The orange bars indicate the heterozygous MF region, while the white bars indicate the homozygous FF region. Several well-known genes located on chromosome 7 are indicated with other papilloma resistance loci, *Skts1, 2*, and genetic markers. Genetic positions shown are according to the Ensembl database (http://uswest.ensembl.org/index.html), the Mouse Genome Informatics Database (http://www.informatics.jax.org/). (B) Comparison of average papilloma numbers/mouse between a congenic line (a) and (b). The top panel shows papilloma incidence in a congenic line (a). The bottom panel shows papilloma incidence in a congenic line (b). The orange bars represent papilloma numbers of MF heterozygous congenic mice. The grey bars represent those of FF heterozygous congenic mice. (C) Photos of representative mice on TPA treatment. Dorsal back skin of MF heterozygous mice of congenic lines (a), (b) and a homozygous FF mouse of a congenic line (a) as a control at 10 weeks after initiation.

Previous QTL analysis identified at least two independent skin tumor susceptibility loci (*Skts1* and *Skts2*) on mouse chromosome 7 [12]. We have recently identified a series of skin papilloma resistance loci, *Stmm* (*S* *kin tumor modifier of MSM*) loci, using F1 backcross mice between a wild derived inbred mouse strain (MSM) and a susceptible inbred mouse strain (FVB/N). Two of these loci are located in the vicinity of *Skts1* and *2* on chromosome 7: *Stmm1* was mapped near the markers D7SNP507 and D7SNP513, and *Stmm2* near the markers D7SNP6 and D7Mit10 [16], (**Figure 1A**). To confirm the presence of low-penetrance susceptibility genes in these regions, we selected resistant F1 backcross mice for further backcrossing to FVB/N mice to generate two N10 congenic mouse lines that span either the *Stmm1* (congenic line a) or the *Stmm2* (congenic line b) region (**Figure 1A**). First, these congenic lines were subjected to the DMBA-TPA skin carcinogenesis experiment, according to the standard protocol. As previously shown, FVB/N mice are highly susceptible to the two-stage skin chemical carcinogenesis. Similarly, homozygous FVB/FVB (FF) mice of congenic lines (a) (n = 13) and (b) (n = 14) were highly susceptible to skin carcinogenesis, developing on average 18 papillomas at 10 weeks after initiation, and about 40 papillomas at 20 weeks after initiation (**Figure 1B, 1C** **and** **Table 1**). In contrast, heterozygous MSM/FVB (MF) mice of a congenic line (a) developed an average of 3.6±3.8 papillomas/mouse at 10 weeks after initiation (n = 15; compared with control: *P* = 3.2^−10^) (**Figure 1B, 1C** **and** **Table 1**), consistent with this region conferring resistance to skin papillomas. On the other hand, MF heterozygous mice of a congenic line (b) had little effect on papilloma development. The number was an average of 12.5±5 papillomas/mouse at 10 weeks (compared with control: *P* = 0.13) and 38.3±8.7 papillomas/mouse at 20 weeks after initiation (n = 14; compared with control: *P* = 0.07) (**Figure 1B, 1C** **and** **Table 1**). In summary, MF heterozygous mice of a line (a), corresponding to *Stmm1*, developed significantly fewer papillomas than FVB/N mice. On the other hand, MF heterozygous mice of a line (b), corresponding to *Stmm2,* developed about the same number of papillomas as control wild type FVB/N mice. These results clearly suggest *Stmm1* has a strong suppressive effect on papilloma development.

**Table 1:** Papilloma incidence in the congenic lines.

Congenic line	Chromosomal region (cM)	10 weeks			20 weeks		
		MF	FF	P-value	MF	FF	P-value
a	1.91–60.49	3.6 (0–14; n = 15)	19.8 (11–26; n = 13)	3.2E-10	17.8 (3–29; n = 15)	46.2 (36–66; n = 13)	1.50E-08
b	68.16–88.85	12.5 (4–21; n = 14)	15.8 (9–29; n = 14)	0.13	36.8 (19–50; n = 14)	44.1 (28–67; n = 14)	0.07
c	20.65–60.49	2.7 (0–13; n = 7)	19.8 (11–26; n = 13)	5.8E-07	10.5 (4–32; n = 7)	46.2 (36–66; n = 13)	2.50E-07
d	43.4–60.49	5.9 (1–11; n = 13)	24.2 (14–44; n = 10)	4.20E-07	26.5 (2–42; n = 12)	45.5 (30–61; n = 8)	0.003
e	49.01–60.49	3.7 (0–11; n = 12)	20.1 (5–44; n = 11)	1.40E-09	16.5 (0–37; n = 12)	36.7 (28–50; n = 10)	0.0003
f	52.92–54.45	2.7 (0–10; n = 9)	18.2 (12–25; n = 6)	4.88E-06	15.125 (2–39; n = 9)	47.25 (36–54; n = 6)	0.0007
g	1.91–20.65	20.2 (11–29; n = 5)	19.8 (11–26; n = 13)	0.87	39.4 (26–45; n = 5)	46.2 (36–66; n = 13)	0.17
h	1.91–51.6	20.5 (16–25; n = 3)	19.8 (11–26; n = 13)	0.31	41.5 (29–54; n = 3)	46.2 (36–66; n = 13)	0.55
i	20.65–54.45	20.8 (7–27; n = 5)	24.6 (21–29; n = 6)	0.31	30.2 (24–37; n = 5)	40.4 (32–49; n = 6)	0.04

Abbrevations: cM, centi-morgan from MGI; N.D., not determined; MF, MSM/FVB; FF, FVB/FVB. *P*-value is from standard *t*-test.

### Narrowing down the Genetic Distance of *Stmm1* on Chromosome 7

**Figure 2:**
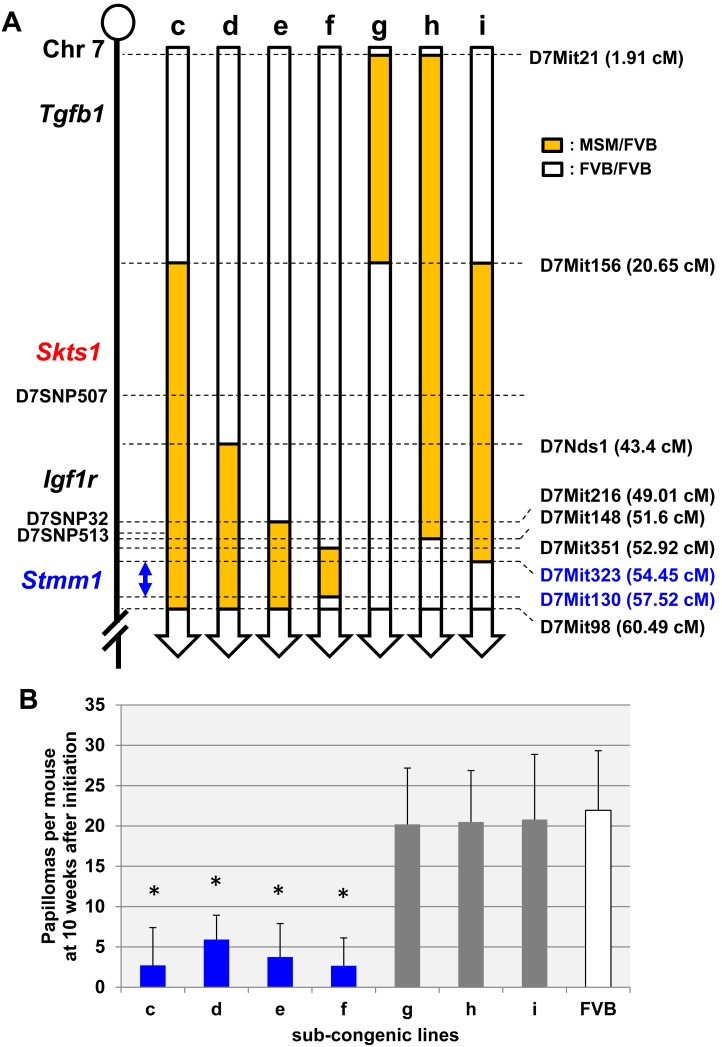
Summary of linkage analysis of sub-congenic lines for *Stmm1* locus on proximal chromosome 7. (A) Each bar represents a different sub-congenic line. The location of the orange and white bars indicates the region on chromosome 7. The orange bars indicate the heterozygous MF region, while the white bars indicate the homozygous FF region. The blue arrowed line indicates the *Stmm1* region narrowed down by congenic mapping. (B) Papilloma incidence of sub-congenic lines c (n = 7), d (n = 13), e (n = 12), f (n = 9), g (n = 5), h (n = 3), i (n = 5), and FVB (n = 36) in 10 weeks after initiation. The blue bars represent congenic lines that retained papilloma resistance, whereas the grey bars represent congenic lines that didn’t show resistance. Error bar represents the standard deviation (S.D.). The *P* value was calculated for papilloma number at 10 weeks by *t*-test (*P<0.01).

On the basis of skin carcinogenesis experiments of lines (a) and (b), we focused on a line (a), which contains the *Stmm1* locus and showed a much stronger suppressive effect on papilloma development. A series of congenic lines (c–i) containing different overlapping regions were generated from mice of a line (a) (**Figure 2A** **and** **Table 1**). These sub-congenic lines were subjected to the DMBA/TPA chemical carcinogenesis protocol and their papilloma development was monitored for a period of 20 weeks (**Table 1**). For each congenic line, we documented the number of papillomas at 20 weeks after initiation.

**Figure 2A and 2B** show a genetic map and papilloma incidence of each congenic line. Of the seven sub-congenic lines (c-i) tested, four lines (c, d, e, and f) showed significant linkage with papilloma incidence (**Figure 2B** **and** **Table1**). By determining the minimum-overlapping region within the four positive congenic lines (c, d, e, and f) and by excluding the regions of the three congenic lines (g, h, and i) that did not show an effect (**Figure 2B** **and** **Table 1**), we were able to narrow down the location of the *Stmm1* region to an interval of about 3 cM (indicated by a blue arrowed line) from 54.54 to 57.52 cM (**Figure 2A and 2B**). This region does not overlap with the previously reported *Skts1* locus. These data suggest that *Stmm1* is a novel skin tumor susceptibility locus on chromosome 7, conferring resistance to papilloma development.

### Allelic Imbalances in Favor of the FVB/N Allele of the *Stmm1* Locus in Skin Tumors from F1 Backcross and Congenic Mice

**Figure 3:**
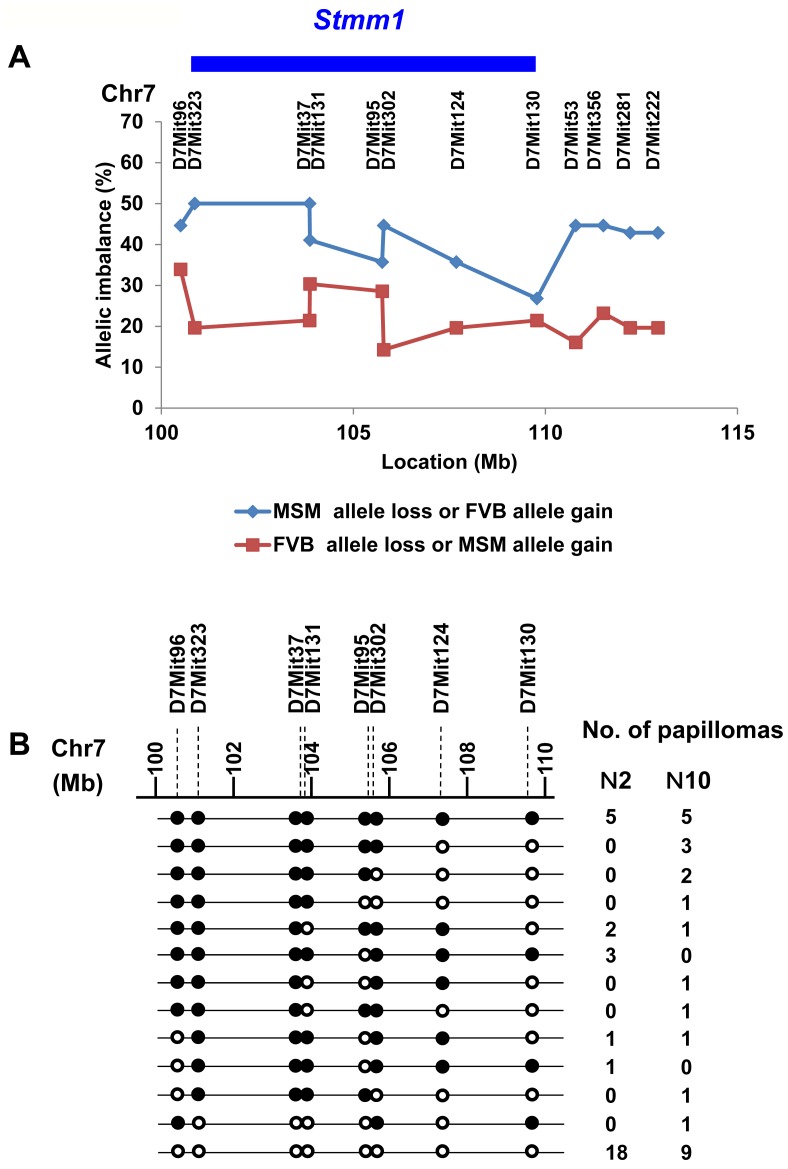
Allelic imbalance analysis of *Stmm1* region on chromosome 7. (A) Frequency of allelic imbalance detected by SSLP analysis on proximal chromosome 7. Data are derived from papillomas of F1 backcross (N2) and N10 congenic mice (N2, n = 30; N10, n = 26). The percentages of chromosome imbalances are plotted for different microsatellite markers (for detailed information, see **Table 2**). The blue bar represents MSM allele loss or FVB/N allele gain. The red bar represents FVB/N allele loss or MSM allele gain. (B) Detailed analysis of imbalance patterns around *Stmm1* region in papillomas. Open circles represent no imbalance; closed circles represent imbalance in favor of the FVB/N allele.

We carried out a detailed investigation of allelic imbalance on chromosome 7 to determine whether somatic changes would provide a more specific localization of *Stmm1*. This allelic imbalance analysis was performed using twelve informative microsatellite markers (**for detailed information, see** **Table 2**). **Figure 3A** shows the frequency of allelic imbalances near the *Stmm1* locus in papillomas from F1 backcross (n = 30) and N10 congenic mice (n = 26). 5/30 and 5/26 papillomas from F1 backcross and N10 congenic mice showed losses of MSM alleles across the whole region, but others exhibited regional losses involving smaller chromosome fragments (**Figure 3B**). Papillomas from N10 congenic mice showed the higher frequency of allelic imbalances than those from F1 backcross mice (17/26 vs 12/30, *P* = 0.0005), probably because other MSM alleles retained in genomic DNA of F1 backcross mice had an influence on the frequency of allelic imbalances in *Stmm1*. Two allelic imbalance peaks were detected within the minimum-overlapping region of 3 cM identified with the multiple congenic lines of *Stmm1* (**Figure 3A and 3B**), one was localized within 3.4 Mb region between D7Mit96 (100.5 Mb) and D7Mit131 (103.87 Mb), the other was within 2 Mb region between D7Mit95 (105.75 Mb) and D7Mit124 (107.68 Mb). When these two regions are combined, the total physical size of the candidate region can be cut down to about 5.4 Mb. This interval still contains a large number of genes. Further congenic and somatic mapping will refine the candidate region to facilitate gene identification.

**Table 2:** Microsatellite markers used for genetic mapping and allelic imbalance analysis.

Chromosome	Name	cM	bp
Chr7	D7Mit21	1.91	3266568–3266693
Chr7	D7Mit156	20.65	34801081–34801224
Chr7	D7Mit18	-	-
Chr7	D7Mit276	34.35	62277634–62277744
Chr7	D7Nds1	43.46	74676373–74676604
Chr7	D7Mit30	45.71	81485676–81485905
Chr7	D7Mit319	46.43	82038932–82039044
Chr7	D7Mit350	47.43	83586089–83586207
Chr7	D7Mit62	48.36	84640886–84641032
Chr7	D7Mit216	49.01	87447195–87447372
Chr7	D7Mit320	49.64	89436896–89436978
Chr7	D7Mit352	50.64	90599957–90600077
Chr7	D7Mit148	51.6	92745007–92745138
Chr7	D7Mit184	52.81	96843943–96844092
Chr7	D7Mit351	52.92	96901062–96901168
Chr7	D7Mit354	53.93	98911768–98911890
Chr7	D7Mit96	54.36	100505707–100505782
Chr7	D7Mit323	54.45	100875936–100876050
Chr7	D7Mit37	54.85	103860643–103860803
Chr7	D7Mit131	54.85	103870606–103870726
Chr7	D7Mit95	55.98	105751411–105751503
Chr7	D7Mit302	55.98	105794470–105794615
Chr7	D7Mit124	57.21	107683097–107683208
Chr7	D7Mit219	57.21	109096065–109096188
Chr7	D7Mit130	57.52	109788134–109788282
Chr7	D7Mit53	57.86	110792641–110792834
Chr7	D7Mit356	58.21	111514329–111514441
Chr7	D7Mit281	58.74	112212330–112212440
Chr7	D7Mit222	59.13	112934466–112934612
Chr7	D7Mit98	60.49	114917267–114917439
Chr7	D7Mit40	62.01	116817442–116817642
Chr7	D7Mit330	64.07	119656690–119656814
Chr7	D7Mit255	68.16	124914439–124914573
Chr7	D7Mit104	71.29	129101439–129101574
Chr7	D7Mit165	72.74	129762283–129762408
Chr7	D7Mit43	73.19	130321003–130321213
Chr7	D7Mit10	81.76	136391134–136391318
Chr7	D7Mit259	88.85	144757504–144757647

### BrdU Chase Experiments using *Stmm1* Congenic Mice

**Figure 4:**
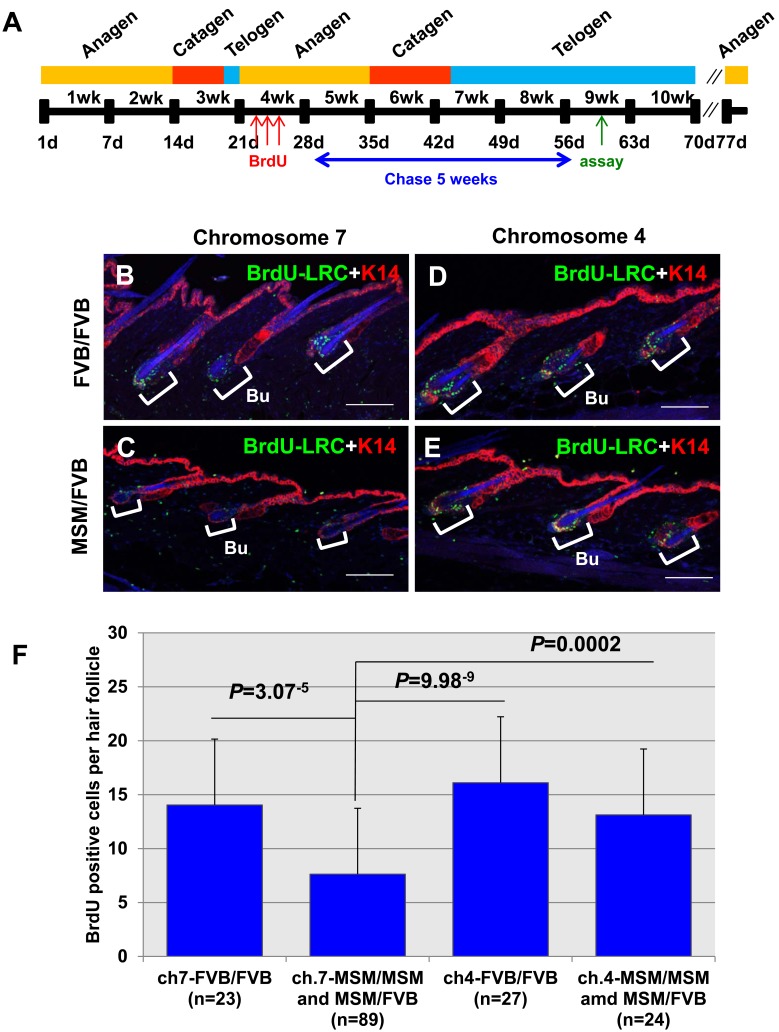
*Stmm1* congenic mice show a decrease of BrdU-LRCs. (A) The timetable of BrdU chase experiment. Multiple color bars indicate the mouse hair cycles (orange, anagen; red, catagen; and blue, telogen) and the bold black line below indicates the postnatal days of mice. (B) Representative double-immunostaining patterns of BrdU-LRCs (green) and Keratin 14 (K14) (red) in the skin from a FF homozygous of a congenic line (a) on chromosome 7, (C) a MF heterozygous mouse of a congenic line (a), (D) a FF homozygous mouse of a congenic line having the linkage region on chromosome 4, and (E) a MF heterozygous mice of a congenic line having the same linkage region on chromosome 4. (F) The numbers of BrdU-LRCs per hair follicle in dorsal back skin sections are plotted. The blue bars represent the number of BrdU-LRCs in each congenic line, FF homozygous mice of a congenic line (a) on chromosome 7 (n = 23), MF heterozygous and MM homozygous mice of a congenic line (a) (n = 89), FF homozygous mice of a congenic line having the linkage region on chromosome 4 (n = 27), and MF heterozygous and MM homozygous mice of a congenic line having the same linkage region on chromosome 4 (n = 24). The *P*-value was calculated for BrdU-LRC number by *t*-test. Error bar represents standard deviation (S.D.). Abbreviation: Bu, Bulge. Scale bars, 100 µm.

In the original report, we classified papillomas into three categories based on their diameter, and carried out linkage analysis for each category. This analysis revealed strong linkage at *Stmm1* and *Stmm2* on chromosome 7 to the total papilloma number, the number of papillomas <2 mm, and the number of papillomas of 2–6 mm in diameter. However, these linkage peaks at *Stmm1* and *Stmm2* completely disappeared when the analysis was confined to papillomas >6 mm. These results suggested that *Stmm1* and *2* genes function only at an early papilloma stage, but are not involved at the later stage of papilloma progression [16]. To gain the insight into the function of *Stmm1* locus, we carried out BrdU-LRC analysis with congenic lines (**Figure 4**). Using a thymidine derivative, such as BrdU, DNA strands of adult stem cells can be labeled at the moment they are synthesized during development. Adult stem cells are quiescent and their DNA strands segregate non-randomly. As such, epidermal stem cells can retain the BrdU label indefinitely during adulthood and are accordingly referred to as label retaining cells (LRCs). The timetable of this BrdU chase experiment is shown in **Figure 4A** (for detailed information, see Material and Methods).

As a result, heterozygous MF mice of a congenic line (a) showed significantly reduced BrdU-LRCs in the bulge of hair follicles, compared with those of homozygous FF mice of a line (a), as a positive control (*P* = 9.98^−9^) (**Figure 4A, 4B, and 4F**). In addition, the same BrdU chase experiment was carried out using heterozygous MF mice of a congenic line having the region of D4Mit17-D4Mit15 on chromosome 4, previously found to confer strong resistance to late stage papillomas (>6 mm in diameter) [16]. Heterozygous MF mice of a chromosome 4 congenic line did not exhibit a change in the number of BrdU-LRCs compared with that of their homozygous FF littermates (**Figure 4C, 4D, and 4F**). These results suggest that gene(s) located within the *Stmm1* locus may have an influence on papillomagenesis step in the two-stage skin carcinogenesis by regulating epidermal quiescent stem cells.

## Discussion

In this study, the location of *Stmm1* was refined to a genetic interval of about 3 cM on proximal chromosome 7 by using multiple congenic lines. By taking advantage of allelic alterations in papillomas from F1 backcross and N10 congenic mice, we further narrowed down the physical interval to about 5.4 Mb. We carried out BrdU chase experiments with congenic lines containing *Stmm1*, and observed a decrease of BrdU-LRCs in a congenic strain that is M/F heterozygous or M/M homozygous for *Stmm1* on chromosome 7. These results suggest that *Stmm1* modifies papilloma development by regulating epidermal quiescent stem cells.

As previously shown, we screened stage-specific papilloma modifier loci on the basis of the size. *Stmm1* and *Stmm2* were identified on chromosome 7 as modifier loci conferring resistance to papillomas of smaller size [16]. They were mapped in the vicinity of *Skts1* and *2* loci, previously identified using a wild-derived inbred mouse strain, *M. spretus* [12]. *Skts1* was narrowed down to around 15 Mb region between D7Mit193 (31.4 cM) and D7Mit248 (35.2 cM), which is proximal to *Igf1r* by congenic analysis [17]. Carcinogenesis experiments using congenic mice presented in this report show that *Stmm1* locus is distal to *Skts1* (**Figure 2A and 2B**). In addition, mice of congenic lines (d, e, and f) having only *Stmm1* region produced almost the same number of papillomas as mice of a congenic line (c) having both *Skts1* and *Stmm1* region did. Congenic lines (h and i) having only *Skts1* region didn’t show any effect (**Figure 2A and 2B**). However, the number of mice in congenic lines (g, h and i) is relatively low (**Table 1**). We cannot completely exclude the possibility that the region of congenic lines (g, h, and i) has additional other *Stmm1* genes. Nevertheless, it seems likely that MSM mice do not have a modifier gene in *Skts1* region and *Stmm1* is independently capable of reducing the risk of developing DMBA-TPA-induced papillomas. In contrast, mice of a congenic line (b) having *Stmm2* showed much weaker effect on papilloma suppression (**Figure 1**). *Stmm2* identified in the vicinity of *Skts2* and *Hras* on chromosome 7 showed the same effect on allele specificity of *Hras* mutation as *Skts2* did, suggesting that the same gene might be responsible for *Skts2* and *Stmm2* [16]. Although carcinogenesis experiments using congenic mice having *Skts2* have not been reported yet, data presented in this report suggests that *Stmm2* and *Skts2* genes may have a small effect on papilloma multiplicity compared with *Stmm1* (**Figure 1A**). However, a congenic line (b) does not fully cover *Stmm2* region. The interval between D7Mit98 (60.49 cM) and D7Mit255 (68.16 cM) is missing in any congenic line tested (**Figure 1A**). Further congenic study will give us the answer for whether *Stmm2* gene is present in this interval in the near future.

Koning *et al*. (2007) showed that low-penetrance susceptibility genes, even when present in a heterozygous state in congenic mice, can influence somatic genetic changes in tumors and that these alterations can be exploited for the rapid fine mapping of putative susceptibility loci [17]. To narrow down *Stmm1* candidate region, we carried out allelic imbalance analysis of papillomas from [(FVB/N×MSM)×FVB/N] F1 backcross and MF heterozygous mice of N10 congenic line (a) (**Figure 3**). As a result, we detected allelic imbalance showing the highest frequency in *Stmm1* region, in spite of the fact that both parental chromosomes carried the same FVB Hras gene as well as other alleles on distal chromosome 7 (**Figure 3**). This clearly shows that one or more low-penetrance susceptibility genes near *Stmm1* locus can affect somatic genetic change in tumors, independently of the effects of *Hras*. Allelic alterations with the highest rate in favor of FVB alleles were detected within the minimum-overlapping region of *Stmm1* congenic lines using the marker D7Mit323 and D7Mit37, which are located at approximately 100.88 Mb and 103.8 Mb, respectively. The physical size of this region showing allelic alterations is about 5.4 Mb. However this region still contains a large number of genes, some of which are already reported to be related to cancer. *Rrm1 (Ribonuclease reductase M1)* gene encodes the regulatory subunit of ribonucleotide reductase, the molecular target of gemcitabine. A SNP in the promoter region of *Rrm1* gene was reported to be associated with progression free survival in non-small-cell lung cancer patients treated with gemcitabine-based chemotherapy [18]. The NUP98 protein has several distinct roles within the nucleus of the normal cell. Fusion of *NUP98* to a large number of partner genes leads to the generation of leukemogenic *NUP98* fusion proteins. *NUP98* fusions are associated with a wide spectrum of hematopoietic malignancies, such as AML [19]. *RhoG* is another important caindidate gene in this region. Rho proteins belong to the Ras superfamily. They are small (21–25 kDa) molecules that share structural homology and become activated when bound to GTP. Rho GTPases have been reported to contribute to most steps of cancer initiation and progression including the acquisition of unlimited proliferation potential, survival and evasion from apoptosis, angiogenesis, and tissue invasion [20]. RhoG was recently reported to be highly expressed in human glioblastoma and mediate glioblastoma cell invasion [21]. The precise functions of these candidate genes in DMBA-TPA induced skin papilloma model remain to be elucidated yet. Further congenic and functional study will be required for gene identification in the near future.

Several lines of evidence come from studies on mouse skin tumor development where the concept that slowly dividing LRCs rather than rapidly proliferating TA (Transit Amplifying) cells are capable to expand during skin tumor promotion is long established [22]. Already in the 1980th it was shown that in mice with [3H] thymidine labeled epidermal LRCs these LRCs scarcely underwent mitosis and remained in the basal layer upon TPA treatment, whereas the proliferating cells dislocated rapidly from the basal layer undergoing terminal differentiation [23]. Furthermore, LRCs of hair follicles retained carcinogen-DNA adducts [24], and even after ablation of cycling cells in the epidermis with a chemotherapeutic drug prior to DMBA treatment, the rate of carcinoma formation was unchanged, indicating that tumor initiation occurred in quiescent (stem cells) rather than rapidly proliferating (TA) cells [25]. On the basis of these concepts, we carried out BrdU-LRC analysis using congenic mice containing *Stmm1* region. Interestingly, mice of a congenic line (a) containing *Stmm1* locus exhibited a significant reduction of BrdU-LRCs in the bulge of hair follicles (**Figure 4**). These results strongly suggest *Stmm1* gene could suppress papilloma formation by altering the behavior of adult epidermal quiescent stem cells in hair follicles. Combination of BrdU labelling, DMBA-TPA carcinogenesis and allelic imbalance analysis using congenic mice may facilitate gene identification step and functional characterization of the gene responsible for *Stmm1*.

## Materials and Methods

### Mice and Tumor Induction

This study was carried out in strict accordance with the recommendations in the Guide for the Care and Use of Laboratory Animals of the Ministry of Education, Culture, Sports, Science, and Technology of Japan. The protocol was approved by the Committee on the Ethics of Animal Experiments of Chiba Cancer Center (Permit Number: 13–18). All efforts were made to minimize suffering. FVB/N mice were purchased from Japan Clea. MSM/Ms mice have been maintained in the Experimental Animal Facility at Niigata University and Chiba Cancer Centre for more than 20 years. In a large F1 backcross study using [(FVB/N×MSM/Ms)×FVB/N], papilloma resistance loci were identified by QTL analysis [16]. Resistant F1 backcross mice were selected for further backcrossing to FVB/N mice over at least 10 generations, ultimately leading to congenic lines (a) and (b) containing MSM/Ms allele of *Stmm1* and *2* on the FVB/N background (**Figure 1A**). Multiple congenic lines containing different overlapping regions were generated from line (a) mice (**Figure 2A**). These congenic mice were treated following the same skin tumor induction protocol, as reported previously [16]. In short, the mice (8–12 weeks) received a single dose of DMBA (25 µg per mouse with 200 µl of acetone) and, starting 1 week after initiation, the animals were subjected to TPA (200 µl of 0.1 mM solution in acetone) twice weekly for 20 weeks. Papilloma numbers were counted every week until 20 weeks after initiation.

### DNA Preparation, Genotyping, and Allelotyping using Microsatellite Markers

DNA was prepared from papillomas, corresponding normal tail tips and kidneys. Microsatellite markers were amplified by standard methods. Each marker’s order and distance were estimated from the Ensembl database (http://uswest.ensembl.org/index.html), the Mouse Genome Informatics Database (http://www.informatics.jax.org/), the NIG Mouse Genome Database (http://molossinus.lab.nig.ac.jp/msmdb/index.jsp), and the Mouse Microsatellite Database of Japan (http://www.shigen.nig.ac.jp/mouse/mmdbj/top.jsp). D7SNP markers and C7. loc64 are described in [16]. To determine susceptibility in congenic lines, unpaired two-tailed Student’s t-test was used. Differences of 50% or more in the intensity ratios of the two alleles in papilloma DNA relative to the corresponding level in kidney as a normal control were defined as allelic imbalance by SSLP (Simple Sequence Length Polymorphisms) analysis, as previously described [16], [17]. Percentages of allelic imbalances in different crosses were compared using 2×2 Chi square test (Fisher’s test).

### BrdU Chase Experiment and Immunohistochemistry

For chase experiments, BrdU (Sigma-Aldrich) was administered by peritoneal injection to postnatal day 24 (P24) mice (40 µg per gram of bodyweight) on 3 consecutive days. Mice were then chased from day 24 to day 59. Chased skin tissues were fixed with 4% paraformaldehyde at 4°C overnight. The endogenous peroxidase activity in the specimens was blocked by treatment with 0.3% H_2_O_2_ and samples were then rinsed with PBS. Sections were incubated with primary antibodies diluted in blocking buffer overnight at 4°C and stained with rat anti-BrdU (1∶100 Abcam) antibody and rabbit anti-keratin 14 (1∶500, Covance Research). Secondary antibodies were Alexa Fluor 488-conjugated anti-rat antibody (1∶100, Molecular Probes, Invitrogen) and Alexa Fluor 568-conjugated anti-rabbit antibody (1∶100, Molecular Probes, Invitrogen). Nuclei were counterstained with Hard Set Mounting Medium with DAPI (Vector). All fluorescence images were obtained with a Leica TCS SPE confocal microscope equipped with a DMI4000B (10X/0.40, 20X/0.70, and 40x/1.25 oil immersion objective).
